# How was the intern year?: self and clinical assessment of four cohorts, from two medical curricula

**DOI:** 10.1186/1472-6920-14-123

**Published:** 2014-06-24

**Authors:** Gillian Laven, Dorothy Keefe, Paul Duggan, Anne Tonkin

**Affiliations:** 1 School of Medicine, Faculty of Health Sciences, the University of Adelaide, Adelaide, South Australia, Australia; 2 School of Paediatrics and Reproductive Health, Faculty of Health Sciences, the University of Adelaide, Adelaide, South Australia, Australia; 3 Medicine Learning and Teaching Unit, Faculty of Health Sciences, the University of Adelaide, Adelaide, South Australia, Australia

**Keywords:** Evaluation/assessment, Clinical performance, Curriculum development/evaluation, Problem-based, Intern/house officer training, Competence

## Abstract

**Background:**

Problem-based curricula have provoked controversy amongst educators and students regarding outcome in medical graduates, supporting the need for longitudinal evaluation of curriculum change. As part of a longitudinal evaluation program at the University of Adelaide, a mixed method approach was used to compare the graduate outcomes of two curriculum cohorts: traditional lecture-based ‘old’ and problem-based ‘new’ learning.

**Methods:**

Graduates were asked to self-assess preparedness for hospital practice and consent to a comparative analysis of their work-place based assessments from their intern year. Comparative data were extracted from 692 work-place based assessments for 124 doctors who graduated from the University of Adelaide Medical School between 2003 and 2006.

**Results:**

Self-assessment: Overall, graduates of the lecture-based curriculum rated the medical program significantly higher than graduates of the problem-based curriculum. However, there was no significant difference between the two curriculum cohorts with respect to their preparedness in 13 clinical skills. There were however, two areas where the cohorts rated their preparedness in the 13 broad practitioner competencies as significantly different: problem-based graduates rated themselves as better prepared in their ‘awareness of legal and ethical issues’ and the lecture-based graduates rated themselves better prepared in their ‘understanding of disease processes’.

Work-place based assessment: There were no significant differences between the two curriculum cohorts for ‘Appropriate Level of Competence’ and ‘Overall Appraisal’. Of the 14 work-place based assessment skills assessed for competence, no significant difference was found between the cohorts.

**Conclusions:**

The differences in the perceived preparedness for hospital practice of two curriculum cohorts do not reflect the work-place based assessments of their competence as interns. No significant difference was found between the two cohorts in relation to their knowledge and clinical skills. However results suggest a trend in ‘communication with peers and colleagues in other disciplines’ (*χ*^2^ (3, N = 596) =13.10, p = 0.056) that requires further exploration. In addition we have learned that student confidence in a new curriculum may impact on their self-perception of preparedness, while not affecting their actual competence.

## Background

**Figure 1:**
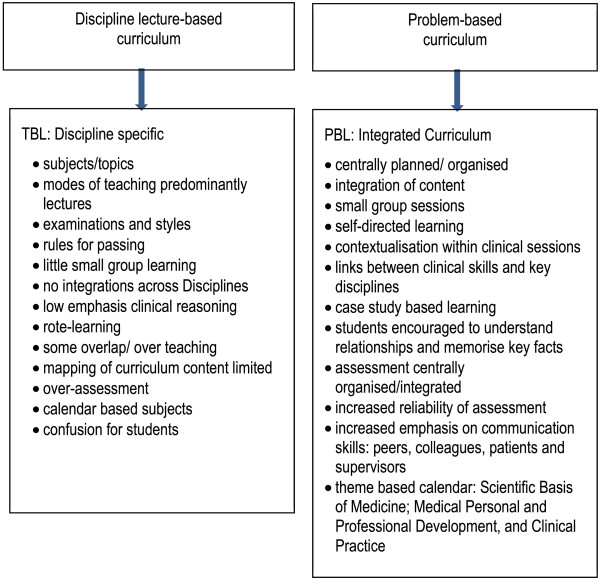
Curriculum differences Discipline lecture-based (TBL) and Problem-based Learning.

In 2000, the University of Adelaide Medical School adopted a new curriculum. This curriculum switch from traditional discipline lecture-based ‘old’ (TLB) to problem-based learning ‘new’ (PBL) as part of a worldwide trend and represented our most significant change since the 1960’s [1]. In the adoption of any new curriculum, it is vital to evaluate its effectiveness, ensuring that standards and quality are maintained or enhanced. The full impact of changes to curricula is not known for some time after graduation, requiring a long-term approach to curriculum evaluation [2]. This study has evaluated how graduates of TLB and PBL curricula perceived their preparedness (self-reflection assessment) for hospital practice after completion of their intern year, in comparison to the work-place based assessment (WPBA) assessment.At the University of Adelaide, years 1–3 in the TLB curriculum were didactic in style, with the program organised into many separate subjects delivered by individual disciplines, primarily in a lecture mode. Years 4–6 were clinically focussed. There was little emphasis on clinical reasoning and relatively little small group learning. The subjects were not integrated in any way with each other, so that a student could be studying the anatomy of the brain, the pharmacology of heart failure, the characteristics of Staphylococcus, and the history of public health all at the same time. Communication skills were delivered in lecture format by staff from psychology, with very little opportunity for students to practise (Figure 1).

The ‘new’ PBL curriculum was centrally planned, with integrated multidisciplinary content, delivered in lectures and student-centred small group (n = 8) sessions. The use of clinical scenarios, designed to encourage students to form links between clinical practice and the basic medical sciences, commenced in 1st year. The scenarios were simple cases of common conditions, and progressed to more complex cases with multiple co-morbidities in 3rd year. Tutors fulfilled primarily a facilitative role and group discussions occupied 6–20 hours per week. There was an increase in emphasis on communication skills (allied health colleagues, patients, peers and supervisors) with opportunities to practise communication skills were introduced using actors, with audio visual recordings for students to review their own performance. Assessment was centrally organised and integrated, and included testing of knowledge and clinical reasoning [3].

In the evaluation of outcomes from an overall curriculum, student satisfaction alone is insufficient [4], and attention must be paid to impacts on student progression, student and graduate satisfaction, career choices or preferences, and career retention.

The effects of different curricula on such an elite group of students, shown to pass examinations irrespective of teaching methods [5], has been relatively inconclusive [1,6,7]. Studies comparing TLB with PBL curricula vary in their findings, from no clear differences in outcomes [1,6,8-10], to observed differences in the areas of social and cognitive dimensions [7], tests of factual knowledge [11], clinical examinations [11,12], and licensing examinations [13].

The outcomes research to date has mainly employed a self-report study design [9,14] and ‘seldom include workplace points of view’ [15]. It is important to widen the focus of evaluation beyond traditional educational outcomes, to include external assessment such as WPBA assessments during the intern year [9,16,17].

In Australia, at the time of this study, all medical graduates spent their first postgraduate year as an ‘intern’, in accredited public hospitals. Throughout Australia, intern assessment processes vary. However, all WPBA are made by senior clinicians and their supervising team and are endorsed by the Medical Board of Australia. In South Australia, an intern has on average five rotations. At the completion of each rotation, the clinical supervisors provide a WPBA report that identifies strengths and weaknesses and gives an overall appraisal of intern performance. The intern end of rotation assessment is ‘high stakes’, however the concept of pass/fail is not used, the intern is assessed as having made satisfactory, borderline or unsatisfactory progress in acquiring intern competencies. If a rotation has not been satisfactory, remedial measures are implemented and progress recorded. A single unsatisfactory rotation will not necessarily need to be repeated if good progress is made during the rest of the year.

The aim of stages I and II of the Medical Graduates Outcomes Program was to follow and compare long-term outcomes of graduates from the two types of curricula: lecture-based (graduates 2003, 2004) and problem-based (graduates 2005, 2006) curricula. To assess how well prepared these graduates felt for their internship and compare this self-assessment with the clinical supervisor-assessment results of their intern year.

## Methods

### Participants, procedure and study design

The cohorts studied graduated from the University of Adelaide Medical program between 2003–2006, with graduates from 2003 and 2004 comprising the TLB cohort, and graduates from 2005 and 2006 comprising the PBL cohort. Methodological triangulation involved data collection via a self-administered questionnaire at the completion of the intern year (one year after graduation), and an audit of intern WPBA reports from five South Australian public hospitals.

Between December 2006 and May 2007 graduates were sent an information pack containing an introduction to the project, a consent form, the Preparedness for Hospital Practice questionnaire and a contact details form to allow data collection for the next two stages of the study. The six month period of contact and follow-up ensured that all graduates had completed their intern year. Graduates who completed their intern year outside of Australia were excluded from this analysis. The audit of intern reports was carried out in June and July 2009 (Audit form available as Additional file 1).

Graduates were asked to rate how well the medical program had prepared them in 13 broad practitioner competencies and 13 areas of clinical and hospital practice using a 5 point Likert scale, from ‘Very well’ through to ‘Not at all well’. The questionnaire was based on two previously validated questionnaires [9,14]. The different areas represent a diverse range of skills and are divided into three sections: Preparation for Hospital Practice (ie history taking and diagnosis); Clinical Skills & Preparedness (ie procedures including consent, prescribing and cannulation), and Resilience (ie level of responsibility and meeting challenges as intern).

The intern audit form was developed based on the structure and content of the WPBA across each hospital. Commonly assessed criteria were identified. The audit was carried out between June and August 2009. Fourteen criteria, ‘Achieving Appropriate Level of Competence’ and an ‘Overall Appraisal’ rating were assessed in the audit. A five point Likert Scale was used to record the competence, from ‘High level of competence’ through to ‘Low competence’.

Ethical approval was obtained from the University of Adelaide Human Research Ethics Committee (H-019-2006 and H-099-2010) and the Ethics Committees of the five public hospitals.

### Analysis

The data were recoded by compression from a 5- to 3-point scale (e.g. ‘strongly agree and agree’, ‘neutral’, and ‘disagree and strongly disagree’). Descriptive statistics (frequencies) were completed for all items by curriculum type. Differences between the curriculum types were examined using separate chi-square tests. In order to account for multiple testing we adjusted for the number of comparisons made (Bonferroni method [18]) to reduce the issue of multiplicity (ie increased rate of type I error). Results presented for each chi-squared test are the adjusted *p*-values.

## Results

A total of 166 graduates (39% of the total number contacted) completed the Preparedness for Hospital Practice Questionnaire (Table 1). Matched WPBA data were available for 124 graduates. The demographics of the responding graduates do not differ significantly from their respective cohort populations for gender (*χ*^2^ (1, N = 458) =0.69, *p* = 0.405). The number of international students from the PBL cohort who responded to the survey was almost double that of the TLB (15 vs 8), but there was no significant difference between the cohorts in terms of domestic or international status (*χ*^2^ (2, N = 165) =4.28, *p =* 0.118). The respondents’ ages ranged from 23–45 years at the time of survey, with 80% (134) of respondents being aged 28–31 years, and no significant difference between the curriculum cohorts (*χ*^2^ (11, N = 165) =11.03, *p* = 0.440). There was no significant difference in the proportion of respondents from each of the 5 hospital training sites (*χ*^2^ (5, N = 166) =5.66, *p* = 0.342) (Table 1).

**Table 1:** Results stages I and II

	**% (N = 423)**
Overall response rate	41.7% (172)*
Preparedness for hospital practice Questionnaire	39.2% (166)*
Intern reports audited with matched data	625 reports (124)
Follow-up consent 3 yr & 10 yr:	37.6% (159)*
Contact details	36.2% (153)*
2003 TLB	24.6% (41)
2004 TLB	23.5% (39)
2005 PBL	24.7% (41)
2006 PBL	27.1% (45)
Female preparedness survey	61.3% (49)
Female intern audit	60.5% (52)

*Not all: graduates completed the questionnaire; consented to the audit or intern reports could be found.

### Respondent self-assessment

**Figure 2:**
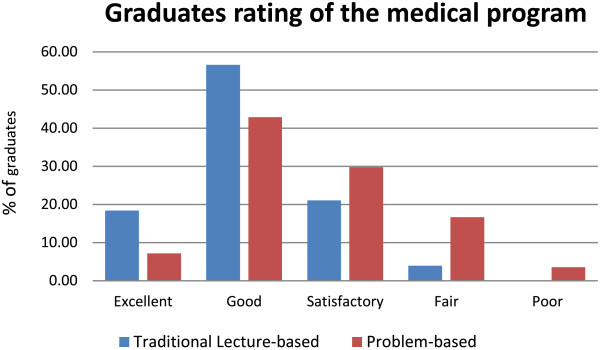
Graduates retrospective rating of the medical program at the completion of their intern year.

For the overall evaluation, graduates from the TLB curriculum were more likely to rate the medical program as ‘excellent/good’ than were the graduates from the PBL curriculum (*χ*^2^ (4, N = 160) =15.55, *p* = 0.004) (Figure 2).

### Preparedness for hospital practice

In two of 13 ‘broad practitioner’ competencies the two cohorts reported significantly different levels of preparedness. The TLB cohort reported higher levels of preparedness for ‘Understanding disease processes’ (*χ*^2^ (4, N = 166) =20.11, *p* < 0.001) while the PBL cohort reported greater preparedness in ‘Being aware of legal and ethical issues’ (*χ*^2^ (4, N = 166) =15.85, *p* = 0.039) (Table 2).

**Table 2:** Graduate self-assessment rating of preparedness of broad practitioner competencies

**How well did the medical program prepare you for......?**	**Curriculum type**	**% More than quite well prepared**	**% Quite well prepared**	**% Less than quite prepared**	***P-value unadjusted***	***P-value adjusted***^**§**^
History taking, clinical examination and selection & interpretation of diagnostic tests	TLB	77.50	20.00	2.50	0.010	0.130
	PBL	60.47	36.05	3.49		
Diagnosis, decision making & treatment including prescribing	TLB	50.00	37.50	12.50	0.613	1.00
	PBL	45.35	39.53	15.12		
Keeping accurate records	TLB	56.25	28.75	15.00	0.444	1.00
	PBL	60.47	31.40	8.14		
Communicating effectively	TLB	76.30	18.75	5.00	0.652	1.00
	PBL	77.90	20.90	1.20		
Working in a team	TLB	67.50	22.50	10.00	0.540	1.00
	PBL	76.70	18.60	4.70		
Being aware of legal and ethical issues	TLB	45.00	32.50	22.50	0.003	0.039*
	PBL	61.60	32.60	5.80		
Managing time effectively	TLB	45.00	28.75	26.25	0.415	1.00
	PBL	40.70	37.20	22.10		
Being aware of own limitations	TLB	72.50	22.50	5.00	0.232	1.00
	PBL	66.30	31.40	2.30		
Understanding disease processes	TLB	67.50	26.25	6.25	<0.001	<0.001**
	PBL	45.35	34.88	19.77		
Understanding the principles of evidence based medicine	TLB	62.50	28.75	8.75	0.422	1.00
	PBL	55.80	32.60	11.60		
Accept the level of responsibility expected of an intern’	TLB	48.75	36.25	15.00	0.891	1.00
	PBL	52.33	36.05	11.63		
Meet the variety of challenges faced’	TLB	48.75	37.50	13.75	0.460	1.00
	PBL	59.30	25.58	15.12		
Dealing with the differing relationships in the hospital context’	TLB	46.25	33.75	20.00	0.250	1.00
	PBL	61.63	29.07	14.46		

^§^post hoc comparisons with Bonferroni correction significant at the *P < 0.05, **P < 0.001.

### Resilience

There was no difference between cohorts for any of the three criteria. The cohorts felt equally prepared to ‘Accept the level of responsibility expected of an intern’ (*χ*^2^ (4, N = 166) = 1.12, *p* = 0.891), ‘Meet the variety of challenges they faced’ (*χ*^2^ (4, N = 166) = 3.62, *p* = 0.460*)* and in ‘Dealing with the differing relationships in the hospital context’ (*χ*^2^ (4, N = 166) = 5.39, *p* = 0.250) (Table 2).

### Clinical skills & preparedness

There were no significant differences between cohorts in the 13 clinical skill competencies (Table 3).

**Table 3:** Graduates self-assessment rating of clinical skills preparedness

***How well did the medical program prepare you for....?***	**Curric--ulum type**	**% More than quite well prepared**	**% Quite well prepared**	**% Less than quite prepared**	***P-value unadjusted***	**P-value adjusted**^**§**^
Basic CPR	TLB	68.75	21.25	10	0.342	1.00
	PBL	74.4	23.3	2.3		
Obtaining valid consent	TLB	50	18.75	31.25	0.008	0.104
	PBL	48.84	38.37	12.79		
Prescribing appropriately	TLB	50	28.75	21.25	0.635	1.00
	PBL	39.5	38.4	22.1		
Writing a prescription	TLB	38.75	32.5	28.75	0.321	1.00
	PBL	43.1	36	20.9		
IV cannulation	TLB	75	17.5	7.5	0.576	1.00
	PBL	69.77	24.42	5.81		
Arterial blood sampling	TLB	53.75	26.25	20	0.996	1.00
	PBL	53.5	24.4	22.1		
Suturing	TLB	53.75	31.25	15	0.388	1.00
	PBL	51.2	27.9	20.9		
Performing an ECG	TLB	37.5	41.25	21.25	0.053	0.689
	PBL	30.2	30.2	39.6		
Administering oxygen therapy	TLB	46.25	35	18.75	0.336	1.00
	PBL	39.53	33.72	26.74		
Correct use of nebuliser	TLB	38.75	28.75	32.5	0.671	1.00
	PBL	31.4	33.7	34.9		
Inserting a nasogastric tube	TLB	38.75	28.75	32.5	0.184	1.00
	PBL	29.07	38.37	32.56		
Urinary catheterisation	TLB	45	33.75	21.25	0.830	1.00
	PBL	40.7	33.7	25.6		
Control of haemorrhage	TLB	40	30	30	0.701	1.00
	PBL	39.5	34.9	25.6		

^§^post hoc comparisons with Bonferroni correction.

### WPBA

The range in number of reports of the interns competence varied from one (three interns) to nine reports (one intern) with the majority of interns having four (n = 24, 18.2%), five (n = 71, 53.8%) and six (n = 25, 18.9%) reports. There were no clear associations of number of reports with cohorts, hospitals, or rotations. A total of 82.0% (N = 533) of reports were signed by the intern, indicating they had received feedback on their rotation.

**Figure 3:**
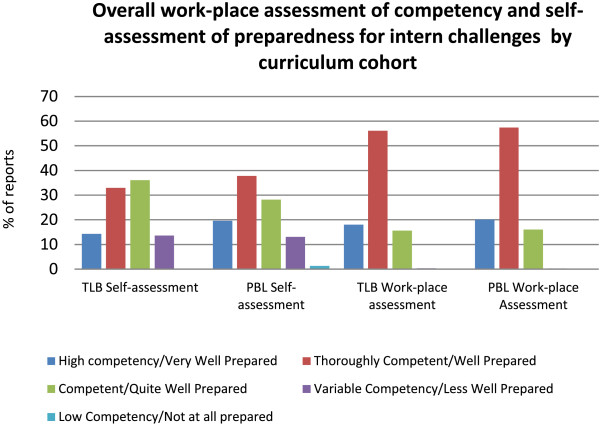
Overall work-place assessment of competency and self-assessment of preparedness for intern challenges by curriculum cohort.

There was no significant difference between curriculum cohorts for ‘Achieving Appropriate Level of Competence’ (*χ*^2^ (1, N = 574) = 1.27, *p =* 0.260) and ‘Overall Appraisal’ (*χ*^2^ (3, N = 615) = 0.22, *p =* 0.974). A comparison of overall appraisal by WPBA and graduates’ self-assessment of preparedness for internship is presented in Figure 3.

A similar pattern is seen for both cohorts, in that graduates assessed themselves more harshly than did their supervisors. Nine (7.3%) graduates received a rating of ‘Variable’ or ‘Low Competence’ on at least one of their individual assessments as an intern. However of these nine only three (2.4%) received an overall rating of ‘Variable Competence’ for that rotation and no graduate received an overall rating of ‘Low Competence’. The low supervisor ratings had no significant relationship to cohort, hospital or rotation.

A comparison of the WPBA assessment of intern competence in 14 skills areas, found only one area where a difference was noted between the two curriculum cohorts (Table 4). There was a trend for graduates of the PBL curriculum to be rated as having higher competence in their ‘Interactions with peers and colleagues from other disciplines’ (*χ*^2^ (3, N = 596) =13.10, p = 0.056).

**Table 4:** Comparison of supervisor-assessment of intern competence in 14 skills areas by curriculum cohort

**Assessed skills & number of reports**	**Competency level**			**Competency level**			***p*- value unadjusted**	***p*- value adjusted**^**§**^
	**TLB curriculum**			**PBL curriculum**				
	**%**			**%**				
	**High/thoroughly**	**Competent**	**Variable**	**High/thoroughly**	**Competent**	**Variable**		
Clinical Assessment/presentation N = 596	83.39	16.23	0.38	87.31	12.69	0	0.185	1.00
Clinical judgement/problem solving N = 614	73.58	26.04	0.38	79.37	19.77	0.86	0.076	1.00
Ongoing management N = 610	81.51	17.74	0.75	86.38	13.04	0.58	0.304	1.00
Documentation N = 596	87.17	12.08	0.75	88.82	11.18	0	0.118	1.00
Physician/patient interactions N = 596	84.9	15.09	0	81.57	18.43	0	0.539	1.00
Senior colleague interactions N = 615	77.74	21.51	0.75	77.71	21.71	0.57	0.931	1.00
Peers & colleagues other disciplines interactions N = 596	86.8	13.21	0	93.65	6.04	0.3	0.004	0.056
Nurses & ancillary staff interactions N = 613	86.41	13.21	0.38	91.38	8.62	0	0.164	1.00
Ethics & integrity N = 593	48.29	51.71	0	44.85	55.15	0	0.106	1.00
Professional skills N = 518	85.61	14.39	0	82.67	17.32	0	0.236	1.00
Theoretical knowledge N = 614	73.86	26.14	0	75.71	24	0.29	0.274	1.00
Learning initiative N = 582	56.59	43.02	0.39	60.19	39.2	0.62	0.543	1.00
Technical competencies N = 580	72.22	27.78	0	71.96	28.05	0	0.931	1.00
Organisational & time management N = 612	78.49	21.13	0.38	81.56	17.87	0.58	0.634	1.00

^§^post hoc comparisons with Bonferroni correction.

## Discussion

We have found that the graduates from both medical curricula were equally competent in their clinical skills as assessed by their clinical supervisors, supporting the findings of previous research [1,6,8-10]. We also found there was a trend for graduates of the PBL curriculum to be rated as better communicators than those from the TLB curriculum. These important communication skills are transferable between clinical settings, research environments and future medical supervisors and teachers. The issues around improved communication skills and team work require further research, ie., does the problem-based curriculum increase these skills or are students today selected for these skills?

Our graduates’ self-assessment of their preparedness for hospital practice varied between curriculum cohorts. PBL graduates self-assessed as being less prepared in two clinical skills (‘Clinical exam & selection and interpretation of tests’ and ‘Understanding disease processes’), while the TLB graduates assessed themselves as less prepared for two of the broader practitioner skills (‘Obtaining consent’ and ‘Legal and ethical issues’). Jones et al. similarly found in a PBL course graduates rated their ability in ‘Understanding disease processes’ less favourably than the TLB graduates [9].

Differences perceived by graduates may be due to differences in student expectations, medical education, the working environment or health care systems [19]. Differences in perception may also relate to specialty bias, for example understanding of disease processes may be more important in an internal medicine rotation than psychiatry. A Kings College School of Medicine and Dentistry survey [20] found that although over 70% of graduates reported their education had satisfactorily equipped them for medical practice, there were significant differences between those in primary care and hospital medicine regarding the relative importance of subjects within the curriculum. However, Ochsmann [19] found deficits in feelings of preparedness irrespective of chosen specialty. The area of how preparedness relates to specialty choice requires further study.

Feelings of preparedness are important in the successful transition from being a student to a practising doctor [19,21]. However, the question of preparedness continues to be an ambiguous one. ‘When junior doctors say they feel prepared, they may not mean they think they are competent’ [22] and it is only by a comparison of self- and supervisor-assessment that we can explore the accuracy of their self–assessment. Our study did not find an association between self- and WPB assessment, supporting Bingham et al’s findings, where trainees assessed themselves more harshly, while their supervisors assessed of trainees as ‘at or above expected level’ for ‘every item in every term’ (43% vs 98.5%) [23]. Qualitative data from the Stage I Preparedness Questionnaire, found two key differences between the TBL and the PBL graduates. The PBL cohorts were much more positive in their responses to how well the program had developed their attitudes to skill development, whilst asking for a greater emphasis on learning basic sciences.

A variety of studies have found that many graduates feel inadequately prepared for the role of junior doctor [24-26] and criticisms that medical schools do not prepare graduates for early medical practice are not new. Goldacre et al. explored UK junior doctors’ views on preparedness in 2010, and found that the level of agreement that medical school had prepared them well for work varied between medical schools and changed over time, ranging from 82% to 30% at one year, to 70% - 27% (respectively) at three year’s post graduation [21].

Both medical schools and medical graduates have questioned preparation and preparedness for early medical practice [21]. Kilminster et al. suggest that the ‘*Emphasis on preparedness (is) misplaced’*[27], and as a result the focus of their work is on exploring the challenges associated with the transition from student to doctor. Interestingly, our graduates from both curricula reported feeling equally well prepared in ‘meeting the challenges’, to ‘accept the level of responsibility’ of an intern, and in ‘dealing with the different relationships in the hospital context’.

Feelings of preparedness may be affected by a number of factors, both internal and external. A comparison of three diverse UK medical schools found that medical graduates’ feelings of preparedness may be affected by individual learning style and personality, but the majority of graduates reported external factors as having the greatest impact [28]. Graduates made reference to external factors such as clinical placements; shadowing and hospital induction procedures and the support of others as important. Illing et al. [28] suggest that perception of preparedness, with respect to external factors, can be addressed by improving hospital induction processes, increased structure and consistency in clinical placements, and addressing perceived weaknesses in clinical procedures identified by the graduates.

There may have been variations in feeling of preparedness from the experience gained during clinical placements in the variety of intern rotations, as ‘institutions and wards have their own learning cultures…’ [27]. However, unlike Illing [28], our study did not demonstrate significant variation between hospitals or rotations, except with respect to the signing of the intern reports and therefore potentially the feedback received by the interns.

There may also be variation in preparedness of the graduates from the two curricula that relate more to their confidence in their learning method. Millan et al. [29] suggest that as graduates are aware of the research purpose, TLB graduates ‘*may overestimate values’* comparing one learning method to another. The graduates we surveyed were aware they were the last two cohorts of the TLB and the first two of the PBL curriculum. The problem-based cohorts may have felt insecure because their curriculum was newly implemented [29] and they may have felt they were missing out on something. This lack of confidence in the PBL cohort may also have been reinforced by some teachers and clinicians who felt disenfranchised and were not fully supportive of the change.

Consideration should also be given when comparing the self-assessment skills of graduates of TLB curriculum with those of PBL, as we may be comparing apples with oranges [30]. Our PBL graduates learned to self-assess using concepts such as pass/fail instead of numerical grades, and may have greater difficulty evaluating their skills [29]. Millan suggests that PBL graduates ‘might view their performance in a different manner’.

### Feedback during internship

The giving and receiving of feedback is important in any training situation, with trainees commonly requesting feedback on their strengths and weaknesses [23]. However, just under 20% of our graduates did not sign their reports (acknowledging feedback). There may be a variety of reasons for this, such as the lack of provision of adequate time for assessment and feedback with the report completed after the intern had left the ‘hospital site’, a lack of training for both medical graduates and supervisors in assessment methods, or possibly a lack of interest in the particular area. In South Australia, demand for some rotations is higher than places available and most interns ultimately undertake rotations that are not within their area of interest, potentially reducing their desire to follow-up on feedback provided. A recent Australian retrospective study of 3390 assessment forms of prevocational trainees found that the forms may underreport performance and do not provide trainees with ‘enough specific feedback to guide professional development’ [23].

### Strengths and limitations

Although the findings reported here are for graduates from one institution’s medical program this may be considered a limitation, however a major strength of this study is the methodological triangulation of two types of data gathered – questionnaire and the audit of intern reports. In addition, each intern was assessed in multiple specialty environments, in one of five large public hospitals, by multiple clinical supervisors, on a range of aspects of clinical knowledge and practice. The range and diversity of the WPBAs thus provides a reliable method of assessment.

Another limitation relates to missing data in the audit of supervisor assessments, which can be traced in the main to two particular rotations: nights and relieving. Comments provided by some supervisors highlight their reluctance to rate interns in these rotations, as they did not observe the interns performing certain skills.

The overall response rate for the longitudinal study of 41.7% may be considered low, but the nature of retrospective longitudinal studies carries with it the inherent issues of loss to follow-up. However, there was no significant difference in the age or gender of our non-responders and responders, and the responders were broadly representative of the four graduating cohorts.

### Future research

The Medical Graduates Outcomes Evaluation Program includes a further 3 stages: ‘Admissions and Selection’, ‘Early (first 5 years)’ and ‘late (10 years)’ postgraduate years. These next stages will provide our university and the broader medical community with a comparison of long-term outcomes between two curriculum cohorts. Our study adds to the body of knowledge that highlights the need for education research in the areas of self-assessment and the giving and receiving of feedback. Curriculum changes based on self-assessment alone run the risk of ‘throwing the baby out with the bathwater’. We suggest that further research is required into the impact of career specialty choices on the perception of how well medical programs prepare their graduates.

## Conclusions

Self- and WPB assessments are both valuable contributors to curriculum evaluation as well as guiding professional development. Our findings demonstrate that the curriculum change from TLB to PBL at our University has ‘*done no harm*’ to our graduates’ clinical practice in the intern year while potentially improving their communication skills and their attitude to skill development. Medical students and graduates, on the whole, are high achieving individuals, who *‘leading up to medical school are groomed and selected for success in a traditional curriculum’* and who would succeed under either curriculum (88).

In addition we have learned that student confidence in a new curriculum may impact on their self-perception of preparedness, while not affecting their actual competence. The transition period from student to intern is a stressful time for all graduates, and it has been reported previously that graduates tend to underestimate when asked to self-assess ‘how well they were prepared for hospital practice’. This perception is not to be discounted, but nor should it be used to support unevaluated curriculum change.

## Abbreviations

(TLB) ‘old’: Traditional (Discipline) lecture-based; (PBL) ‘new’: Problem-based learning; (WPBA) assessment: Work-place based assessment.

## Competing interests

The authors declare that they have no competing interests.

## Authors’ contributions

DK and AT conceived and discussed the scope and design of the longitudinal evaluation project. DK, GL, AT, and PD contributed to the conception and design of stage II. GL conducted the searches, administered the questionnaire Stage I, conducted the audit Stage II and discussed the strategies used with DK. DK, GL, and AT, were jointly involved in the interpretation and data analysis. GL led the writing of the paper and each author contributed significantly to multiple subsequent revisions. All authors approved the final version of the manuscript submitted.

## Pre-publication history

The pre-publication history for this paper can be accessed here:

http://www.biomedcentral.com/1472-6920/14/123/prepub

## Supplementary Material

Additional file 1 Intern audit form 2007_final.pdf. Click here for file
