# Supplementary material for: How was the intern year?: self and clinical assessment of four cohorts, from two medical curricula
Source: BMC Med Educ. 2014 Jun 24;14:123. doi: 10.1186/1472-6920-14-123 (PMC4081487; doi:10.1186/1472-6920-14-123)
Supplement: Additional file 1 — Intern audit form 2007_final.pdf. [file 1472-6920-14-123-S1.pdf]

# MBBS Graduate Outcomes Evaluation

THE UNIVERSITY OF ADELAIDE AUSTRALIA

## Intern Assessment Audit Form

1. ID___________________

2. Year of MBBS Graduation

| | | | |
|---|---|---|---|
| 2003 | **3** | 2005 | **5** |
| 2004 | **4** | 2006 | **6** |

3. Date of Birth __/__/____

4. **Hospital** Intern Assessment **Report Date**: __/__/____

3. **Hospital** at which Intern Year undertaken:

| | | | |
|---|---|---|---|
| Royal Adelaide Hospital | **1** | Modbury Hospital | **4** |
| Lyell McEwin Hospital | **2** | Flinders Medical Centre | **5** |
| The Queen Elizabeth Hospital | **3** | | |

4. **Assessment** of Intern: 1 High competency – 5 Low competency 0=missing information

a) Clinical Assessment/ Presentation _____

b) Clinical Judgement/ Problem Solving _____

c) Ongoing Management _____

d) Documentation _____

e) Physician/Patient Interactions _____

f) Interactions with Senior Colleagues _____

g) Interaction with Peers And Colleagues in other Disciplines _____

h) Interaction with Nurses & Ancillary Staff _____

i) Ethics and Integrity _____

j) Professional Skills _____

k) Overall Appraisal _____

l) Theoretical Knowledge: _____

m) Learning Initiative _____

n) Technical Competencies _____

o) Organisation and Time Management Skills _____

5. Appropriate level of competence achieved: Yes **1** No **0**

6. Progress towards registration:
   Satisfactory **1** Borderline **2** Unsatisfactory **3**

7. General Comments Yes **1** No **0**
   TXT

8. Intern Comments Yes **1** No **0**
   TXT
